# Effect of the Interaction between Seaweed Intake and *LPL* Polymorphisms on Metabolic Syndrome in Middle-Aged Korean Adults

**DOI:** 10.3390/nu15092066

**Published:** 2023-04-25

**Authors:** Junkyung Kwak, Gayeon Hong, Kyung Ju Lee, Choong-Gon Kim, Dayeon Shin

**Affiliations:** 1 Department of Food and Nutrition, Inha University, 100, Inha-ro, Michuhol-gu, Incheon 22212, Republic of Korea; oppo9012@naver.com (J.K.); ghdrkdus215@naver.com (G.H.); 2 Department of Women’s Rehabilitation, National Rehabilitation Center, 58, Samgaksan-ro, Gangbuk-gu, Seoul 01022, Republic of Korea; drlkj4094@korea.kr; 3 Marine Ecosystem Research Center, Korea Institute of Ocean Science and Technology, 385, Haeyang-ro, Yeongdo-gu, Busan 49111, Republic of Korea; kimcg@kiost.ac.kr

**Keywords:** metabolic syndrome, *LPL* gene variants, seaweed, laver, kelp, sea mustard

## Abstract

This study aimed to examine the effect of the interaction between seaweed (laver, kelp, and sea mustard) intake and lipoprotein lipase gene (*LPL*) rs17482735 genotypes on the incidence of metabolic syndrome (MetS). The Korean Genome and Epidemiology Study (KoGES) data of Korean adults aged 40–69 years were used in this study. Information on seaweed intake was obtained from the food frequency questionnaire. To investigate the interaction between seaweed intake and *LPL* rs17482735 genotypes on the incidence of MetS, multivariable Cox proportional hazard models were used after adjusting for confounding variables. There was no significant association in women, but men with TG and TT genotypes of rs17482753 had lower incidence of MetS (HR 0.83, 95% CI 0.71–0.95, *p*-value = 0.01), low HDL-cholesterol levels (HR 0.81, 95% CI 0.69–0.95, *p*-value = 0.01), high triglyceride levels (HR 0.83, 95% CI 0.70–0.99, *p*-value = 0.0471), and high blood pressure (HR 0.79, 95% CI 0.67–0.93, *p*-value = 0.004). Furthermore, the incidence of MetS was lower in men with the highest laver and total seaweed intake and TG and TT genotypes of rs17482735 (HR 0.60, 95% CI 0.43–0.84; HR 0.57, 95% CI 0.41–0.79, respectively). High seaweed intake was negatively associated with MetS, suggesting that *LPL* genetic variations, particularly in men, may be helpful in preventing MetS. These results demonstrate that seaweed intake considering *LPL* genotypes may be beneficial for preventing and treating MetS.

## 1. Background

Metabolic syndrome (MetS) is a collection of closely related vascular risks. These risk factors include excessive visceral fat, hyperlipidemia, glucose intolerance, and hypertension [1]. In a meta-analysis by Hui et al., mortality rates have been reported to be 46% higher among individuals with MetS than those among individuals without MetS [2]. The presence of more MetS risk factors linearly increases mortality from cardiovascular disease (CVD) and coronary heart disease [3]. MetS is caused by risk factors, including obesity, drinking, family history, low physical activity, westernized eating habits, menopausal conditions, and increased age [4]. In particular, interactions between genetic and acquired factors, including diet and physical activity levels, have emerged as important risk factors in MetS [5].

The prevalence of MetS tends to continue to increase until the age of 60 years, as the age bracket increases by 10 years in Asian Indians aged ≥20 years [6]. MetS in individuals ranging from 43 to 79 years of age causes cognitive dysfunction, mainly due to insulin resistance, and decreases recall and intellectual function [7]. In addition, MetS in middle-aged individuals is associated with an increased incidence of depressive symptoms [8]. According to health examination statistics from the National Health Insurance Corporation, the prevalence of MetS among middle-aged Korean adults (40–69 years) in 2020 was 24.4%; 27.7% in men and 20.4% in women [9]. When comparing the prevalence of MetS by age group (40s, 50s, and 60s) in Korea, the prevalences were 16.7%, 21.5%, and 35.0%, respectively [9]. The prevalence of MetS has increased since the 1960s [9].

The lipoprotein lipase (*LPL*) gene encodes *LPL* in adipose tissue, muscle, and heart [10]. *LPL* is an enzyme essential for lipid metabolism [11]. High activity of *LPL* increases high-density lipoprotein cholesterol (HDL-C) levels and decreases triglyceride (TG) and low-density lipoprotein cholesterol (LDL-C) levels [12,13]. On the other hand, *LPL* deficiency causes impaired glucose tolerance, insulin resistance, and increased lipid levels [14,15]. In other words, *LPL* is a critical factor in the development of MetS [16]. Patients with MetS exhibit lower *LPL* activity levels than normal subjects [17].

Previous research suggests that an imbalance in the activity of *LPL* could increase the risk of developing MetS [18]. Mice with overexpressed *LPL* in their skeletal muscles accumulate TG in their muscles, which, in turn, causes insulin resistance. This insulin resistance leads to an increased partitioning of lipids to other tissues, ultimately resulting in obesity [19]. *LPL* is expressed in macrophages and smooth muscle cells, which makes it present in the vessel wall. In this context, *LPL* is thought to contribute to the accumulation of lipids in these cells [20]. In particular, *LPL* rs17482753 was associated with blood lipid levels [21,22]. According to a genetic study of a variety of populations, including East Asians, the *LPL* rs17482753 G allele (major allele) is associated with increased TG and cholesterol levels and decreased HDL-C levels [21]. According to genetic studies in a variety of populations, including East Asian and South Asian populations, decreased TG and increased HDL-C levels are associated with the *LPL* rs17482753 T allele (minor allele) [22]. Therefore, in *LPL* rs17482753, the major allele increases lipid levels, and the minor allele decreases lipid levels [21,22].

Seaweed intake has been reported to help prevent MetS in several studies [23,24,25,26,27,28,29]. In a 4-week placebo-controlled study, seaweed fucoxanthin supplementation (1 mg/day) decreased waist circumference (WC) and fat mass in obese Japanese individuals aged 20–59 years [23]. In addition, fucoxanthin supplementation (3 mg/day) decreased visceral fat, body mass index (BMI), and weight [23]. In a 1-month placebo-controlled study, seaweed powder supplementation (6 g/day) decreased systolic blood pressure and WC in 30 patients in Ecuador [24]. In addition, a study conducted in Koreans found a negative link between seaweed intake and MetS and its components [25,26,27]. In a 4-week clinical trial, seaweed supplementation (48 g/day) enhanced HDL-C levels and enzyme activities that inhibit oxidation and decreased TG and blood glucose levels in Korean patients with type 2 diabetes [25]. Laver intake was inversely associated with abdominal obesity in Korean men and women [26]. In a cross-sectional study, dietary algae intake decreased the risk of diabetes in Korean adults (OR 0.66, 95% CI 0.43–0.99) [27]. Animal studies have shown a negative association between seaweed intake and MetS [28,29]. Carrageenan supplementation in *Sarconema filiforme* (red seaweed) decreased visceral fat, weight, blood pressure, and TC levels in male Wistar rats [28]. Supplementation of fucoxanthin and axanthophyll carotenoids in macroalgae increased HDL-C levels and decreased blood glucose, TG, and glycated hemoglobin levels in C57BL/6N mice [29]. Therefore, seaweed intake has been shown to decrease blood pressure and blood glucose and lipid levels [23,24,25,26,27,28,29].

Previous studies have examined the independent effects of seaweed intake and *LPL* gene variations on MetS. However, the interaction between seaweed intake and *LPL* variants in MetS has not yet been investigated. Therefore, we aimed to identify the interaction between seaweed intake and *LPL* genetic variation in MetS in middle-aged Korean adults.

## 2. Methods

### 2.1. Data Source and Study Participants

**Figure 1:**
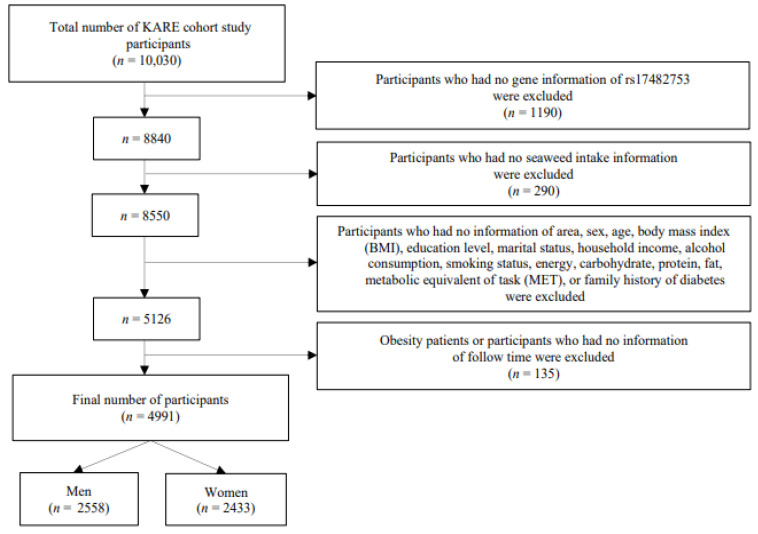
Process flow diagram outlining the steps involved in this analysis.

The Korean Genome and Epidemiology Study (KoGES) Ansan and Ansung data of 10,030 Korean adults aged 40–69 years were used in this study. The KoGES aims to study the public health and disease epidemiology of both rural and urban Korean populations to identify the risks of diseases. The participants were requested to have their lifestyle (alcohol intussusception, smoking, diet, and activity level), sociodemographic status, mental stress, and family history assessed by the interviewer [30]. Of the 10,030 participants, those without SNP rs17482753 gene information were excluded (*n* = 1190), as were those without seaweed intake information (*n* = 290). Of the remaining 8550 participants, those without information on age, sex, area, BMI, marital status, alcohol intussusception, education level, smoking status, family history of diabetes, and metabolic equivalent of task (MET) were similarly excluded (*n* = 3424). Participants with no information regarding follow-up periods were also excluded (*n* = 135). Ultimately, this study consisted of 4991 participants (2558 men and 2433 women) (Figure 1).

### 2.2. Metabolic Syndrome Definition

MetS was evaluated using the National Cholesterol Educational Program Adult Treatment Panel III (NCEP-ATP III) for Asians, which is more suitable for Koreans than other standards and has been used for the clinical diagnosis of MetS [31]. For instance, Asians have a smaller physique than Caucasians; therefore, in the case of abdominal obesity, a lower WC standard is applied [32]. MetS was diagnosed when 3 or more of the following 5 immediate risk constituents were present: (1) blood pressure ≥130/85 mmHg or taking antihypertensive medications; (2) fasting blood glucose ≥110 mg/dL (fasting blood glucose ≥100 mg/dL revised in 2005) or taking antidiabetic medications; (3) HDL-C levels <40 mg/dL (men) and <50 mg/dL (women); (4) TG levels ≥150 mg/dL; and (5) WC ≥90 cm (men) and ≥85 cm (women) [32]. The MetS patients at baseline examination were excluded and only MetS patients in the 2nd to 7th follow-up survey were included.

### 2.3. Assessment of Seaweed Consumption

Dietary information was obtained by trained interviewers using a semi-quantitative food frequency questionnaire (SQ-FFQ). Detailed information regarding the SQ-FFQ has been presented in previous studies [33,34]. The SQ-FFQ contains 106 items that are frequently consumed by Koreans aged 40–69. Seaweed consumption was assessed by the intake of laver, kelp/sea mustard, and total seaweed. Total seaweed intake was the sum of the laver and kelp/sea mustard intake. The standard amount of laver per intake was one sheet (large), and that of kelp/sea mustard was one bowl of soup. The intake frequency was categorized into 9 categories (i.e., none, almost once a month, 2–3 times a month, 1–2 times a week, 3–4 times a week, 5–6 times a week, once a day, twice a day, and thrice a day). The portion size of each food item was categorized as small (half the standard portion size), medium (one standard portion size), or large (two standard portion sizes). In this study, seaweed intake was converted to a daily frequency and multiplied by the number of standard amounts reported for each food item. Then, the study participants’ average daily intakes (g) of laver, kelp/sea mustard, and total seaweed was calculated.

### 2.4. Genotyping and Imputation

SNPs were selected based on the Korean Association Resource (KARE) genotype data, using data collected by the KARE project. Korean genome data were obtained using an Affymetrix genome-wide human SNP array 5.0 (Affymetrix Inc., Santa Clara, CA, USA). In this study, *LPL* rs17482753 at chromosome 8p21.3 (8:19975135) was selected because of its association with HDL-C and TG levels [21,22].

### 2.5. Statistical Analysis

Genetic analysis of *LPL* rs17482753 (minor allele, T) was performed using the GG, TG, or TT genotypes in PLINK (version 1.90 beta). Based on the total seaweed, laver, and sea mustard/kelp intake, participants were classified into four groups. Categorical and continuous variables were compared for the general characteristics of participants using chi-squared tests and *t*-tests, respectively. The participants were separated into two groups, GG and TG or TT, according to their genotype. In this study, we used age, sex, area, marital status, body mass index (BMI), alcohol intussusception, smoking status, education level, family history of diabetes, and MET as adjustment variables. The 95% confidence intervals (CIs) and hazard ratios (HRs) were assessed using a multivariable Cox proportional hazards model. The data were analyzed using SAS version 9.4 software (SAS Institute, Cary, NC, USA). In each test, variables with a *p*-value of <0.05 were considered significant.

## 3. Results

Table 1 shows the participants’ characteristics according to the status of MetS (men with MetS, *n* = 1075; men without MetS, *n* = 1483; women with MetS, *n* = 1066; and women without MetS, *n* = 1367). Both men and women with MetS exhibited statistically significant differences, with a higher BMI, Ansung resident rate, and single rate than those without MetS (all *p* < 0.05). The rate of alcohol intake, current smoking, and family history of diabetes were higher in men with MetS than those in men without MetS (all *p* < 0.05). Women with MetS had higher average age, education level of elementary school or lower, carbohydrate intake, MET, and lower fat intake compared to women without MetS (all *p* < 0.05).

Table 2 shows the participants’ characteristics according to *LPL* rs17482753 genotypes. In men, the TG or TT genotype group had a lower current smoking rate, MET, and a higher family history of diabetes than the GG genotype group (all *p* < 0.05). The average age and single rate of the TG or TT genotype group in women were higher than those of the GG genotype group (all *p* < 0.05).

The association between total seaweed intake and MetS incidence is presented in Table 3. Men with the highest total seaweed intake had a lower incidence of MetS than those with the lowest seaweed intake (Quartile 4; hazard ratio [HR] 0.82, 95% CI 0.69–0.99). Women with seaweed intake in Quartile 2 had a lower incidence of MetS than women with the lowest laver intake (Quartile 2; HR 0.83, 95% CI 0.70–0.98).

The association of laver and sea mustard/kelp intake with MetS is shown in Supplementary Tables S1 and S2. Men with the highest laver intake had a lower incidence of MetS than men with the lowest laver intake (Quartile 4; HR 0.83, 95% CI 0.69–0.99). There was no relationship between sea mustard/kelp intake and MetS in men. In women, there was no association between laver and sea mustard/kelp intake and MetS.

Table 4 shows the association between *LPL* rs17482753 genotypes and MetS incidence. Men with the TG or TT genotype had decreased MetS incidence, low HDL-cholesterol, high triglyceride, and high blood pressure (HR 0.83, 95% CI 0.71–0.95; HR 0.81, 95% CI 0.69–0.95; HR 0.83, 95% CI 0.70–0.99; HR 0.79, 95% CI 0.67–0.93, respectively). No association was found between the *LPL* rs17482753 genotype and MetS in women. In women, there were no associations between *LPL* rs17482753 genotypes and MetS components.

The association between *LPL* rs17482753 genotypes and MetS incidence stratified according to quartile of seaweed intake is shown in Table 5. The MetS incidence decreased for men with the TG and TT genotypes compared to men with the GG genotype in the highest quartile of total seaweed intake (HR 0.57, 95% CI 0.41–0.79). The MetS incidence decreased in women with the TG and TT genotypes than in women with the GG genotype in terms of total seaweed intake (Quartile 2; HR 0.66, 95% CI 0.49–0.89).

Supplementary Tables S3 and S4 show the associations between *LPL* rs17482753 genotypes and the incidence of MetS stratified by quartiles of laver and sea mustard/kelp intake. Compared to men with the G allele, men with the highest quartile of laver intake decreased the MetS incidence in men with the TG or TT genotype (HR 0.84, 95% CI 0.43–0.84). There was no association between *LPL* rs17482753 genotypes and MetS in men stratified by sea mustard/kelp intake. In women, there were no associations between *LPL* rs17482753 genotypes and MetS stratified by laver and sea mustard/kelp intake.

## 4. Discussion

We found a causal-effect relationship between seaweed intake and MetS by LPL gene variations using the prospective cohort study design. In men with the TG or TT gen-otype, the MetS incidence was 43% lower in those in the highest quartile of seaweed intake than that in those in the lowest quartile of seaweed intake. In women with the TG or TT genotype, the MetS incidence was 34% lower in those in quartile 2 of seaweed intake than that in those in the lowest quartile of seaweed intake.

Consumption of total seaweed, including laver, kelp, and sea mustard, was negatively associated with MetS in this present study. In men with the highest total seaweed, intake was significantly associated with a decreased incidence of MetS compared with that in men with the lowest seaweed intake. In line with previous findings, a cross-sectional study found that seaweed intake decreased MetS incidence in middle-aged men (OR 0.70, 95% CI 0.54–0.92) [26]. In a prospective cohort study of 2588 postmenopausal women, seaweed intake and dietary iodine decreased the incidence of MetS (HR  0.52, 95% CI 0.39–0.69; HR 0.61, 95% CI 0.47–0.78, respectively) [35]. A randomized case–control study for 8 weeks revealed that wakame (*Undaria pinnatifida*) intake (5 g/day) in brown algae significantly decreased blood pressure and hypercholesterolemia risk in 36 older Japanese individuals with hypertension [36]. A randomized, double-blind, and placebo-controlled intervention that advanced throughout the 8 weeks showed that iodine-reduced kelp (*Laminaria japonica*) powder supplementation decreased serum LDL-C levels in 50 overweight Japanese adults [37]. Furthermore, in an experimental animal study, the administration of seaweed-protein-derived hydrolysates decreased systolic blood pressure in 20-week-old spontaneously hypertensive rats [38]. Our findings and those of previous studies indicate that seaweed intake may help prevent MetS by reducing blood pressure and lipid levels [26,35,36,37,38]. Peptides sourced from seaweed have demonstrated strong antihypertensive effects by inhibiting ACE, and displayed additional benefits such as lowering cholesterol levels, reducing hyperglycemia, and acting as antioxidants [39]. Furthermore, calcium alginate (Ca-Alg) from seaweed reduced blood cholesterol levels in rats fed a high-cholesterol diet. According to that study, Ca-Alg reduced blood cholesterol levels, possibly due to enhanced fecal excretion of bile acid caused by reduced intestinal reabsorption. This suggests that the reabsorption of bile acids by Ca-Alg could be an effective mechanism for reducing plasma cholesterol levels [40].

A significant association was found between the *LPL* rs17482753 genotype and MetS in this present study. Men with the TG and TT genotypes exhibited a decreased MetS incidence, high TG levels, low HDL-C levels, and high blood pressure compared to men with G alleles. In genetic studies of various populations, including Asians, the *LPL* gene was associated with MetS [41], low HDL-C levels [42], high blood pressure [43,44], diabetes [45,46], abdominal obesity [47], and high TG levels [48]. The T allele (minor allele) of *LPL* rs17482753 is associated with increased HDL-C (Beta 4.97, *p*-value 2 × 10^−41^) and decreased TG levels (Beta 4.97, *p*-value 6 × 10^−39^) [22]. However, in our study, no association was found between the *LPL* rs17482753 genotype and MetS in women. In a previous study, *LPL* rs17482753 and blood lipid levels were investigated in populations including East Asians [22], but differences in sex and age were not investigated. We investigated the association between *LPL* rs17482753 genotypes and MetS by sex. The difference in findings due to sex may be partially due to the fact that estrogen prevents visceral obesity by increasing *LPL* activity in women, whereas testosterone reduces visceral *LPL* activity in men [49]. Although estrogen levels decrease after menopause [49], this study included women aged 40–69 years, and non-postmenopausal women may have been included. Since the average age of menopause in Asia ranges from 42.1 to 49.5 years [50], women under 42 years of age are unlikely to be menopausal.

This study has several strengths. First, we identified the influence of seaweed intake on MetS and effects of the interaction between *LPL* genes and seaweed intake on MetS. Second, we assessed the inverse association between seaweed intake and the incidence of MetS, presenting conclusive evidence of a cause–effect relationship. Third, we investigated sex-specific differences in the association between the *LPL* gene and MetS. Our results showed an inverse association between MetS and *LPL* rs17482753 genotypes in men, but not in women. Therefore, we propose that seaweed intake in men with specific genes can prevent MetS. Fourth, we accounted for confounding factors as much as possible by adjusting for variables such as age, area, smoking, education level, BMI, family history of diabetes, and MET.

Despite these strengths, this study has several limitations. First, because only dietary information from the basic survey was used, there is a possibility of dietary change during this study periods. Second, since we targeted middle-aged Korean adults, these results cannot be applied to other age groups or races/ethnicities. Third, since seaweed intake alone was investigated, it was not possible to identify a mechanism for the effects of specific seaweed nutritional components on MetS. However, based on the Ansan–Ansung Cohort Study of the KoGES data, we observed a significant association between the *LPL* rs17482753 genotype, MetS, and seaweed consumption. We compared men and women with the TG and TT genotypes to those with the G allele and found an association between MetS and seaweed consumption, suggesting that the *LPL* rs17482753 genotype and seaweed intake may have an impact on MetS.

## 5. Conclusions

Our findings showed that the interaction between seaweed intake and *LPL* gene variation decreased the incidence of MetS. In men with the T allele, laver and total seaweed intake decreased MetS incidence. In women with the T allele, the second quartile of total seaweed intake (0.7–1.5 g/day) was associated with a decreased incidence of MetS. Considering *LPL* genotypes and sex, seaweed intake may be beneficial for preventing and treating MetS.

## Figures and Tables

**Table 1:** General characteristics of study participants according to metabolic syndrome.

Variable	Men (*n* = 2558)			Women (*n* = 2433)		
	No MetS	MetS	*p*-Value ^(1)^	No MetS	MetS	*p*-Value ^(1)^
Participants, *n*	1483	1075		1367	1066	
rs17482753 frequency			0.11			0.74
GG (*n* = 3785)	1114 (75.1%)	837 (77.9%)		1027 (75.1%)	807 (75.7%)	
TG, TT (*n* = 1206)	369 (24.9%)	238 (22.1%)		340 (24.9%)	259 (24.3%)	
Age (years)	51.5 ± 9.1	51.2 ± 8.5	0.37	48.6 ± 8.0	52.8 ± 8.6	<0.0001
Area			0.0027			<0.0001
Ansung	580 (39.1%)	484 (45.0%)		430 (31.5%)	624 (58.5%)	
Ansan	903 (60.9%)	591 (55.0%)		937 (68.5%)	442 (41.5%)	
Body mass index (kg/m²)	22.7 ± 2.5	24.5 ± 2.5	<0.0001	23.2 ± 2.7	24.9 ± 2.8	<0.0001
Education level			0.52			<0.0001
≤Elementary school	287 (19.4%)	206 (19.2%)		359 (26.2%)	489 (45.9%)	
Middle school	320 (21.6%)	218 (20.3%)		352 (25.8%)	279 (26.1%)	
High school	538 (36.2%)	420 (39.0%)		522 (38.2%)	248 (23.3%)	
≥College	338 (22.8%)	231 (21.5%)		134 (9.8%)	50 (4.7%)	
Alcohol consumption (g/day)	16.7 ± 26.8	19.5 ± 26.8	0.01	1.4 ± 4.9	1.2 ± 4.4	0.37
Energy (kcal/day)	1994.4 ± 573.0	1991.8 ± 546.1	0.91	1852.1 ± 583.4	1890.0 ± 634.9	0.14
Carbohydrate (g/day)	345.2 ± 93.4	344.2 ± 91.8	0.79	325.1 ± 100.1	340.9 ± 112.9	0.0003
Protein (g/day)	68.3 ± 25.6	68.8 ± 24.1	0.60	64.0 ± 24.6	62.9 ± 26.5	0.30
Fat (g/day)	35.3 ± 18.6	35.2 ± 17.7	0.88	31.2 ± 16.8	28.6 ± 17.5	0.0002
Smoking status			0.02			0.44
Never	315 (21.2%)	183 (17.0%)		1316 (96.2%)	1015 (95.2%)	
Past	451 (30.4%)	325 (30.3%)		13 (1.0%)	13 (1.2%)	
Current	717 (48.4%)	567 (52.7%)		38 (2.8%)	38 (3.6%)	
MET (hours/day) ^(2)^	24.4 ± 15.0	25.0 ± 15.2	0.35	20.9 ± 12.6	23.6 ± 15.0	<0.0001
Family history of diabetes			0.0027			0.22
Yes	133 (9.0%)	136 (12.7%)		179 (13.1%)	122 (11.4%)	
No	1350 (91.0%)	939 (87.3%)		1188 (86.9%)	944 (88.6%)	
Marital status			0.04			<0.0001
Single	47 (3.2%)	51 (4.7%)		132 (9.7%)	163 (15.3%)	
Married	1436 (96.8%)	1024 (95.3%)		1235 (90.3%)	903 (84.7%)	

Data are presented as means ± standard deviation (SD) or number (%). ^(1)^ *p*-values were calculated using the chi-squared test for categorical variables and the *t*-test for continuous variables. ^(2)^ MET, metabolic equivalent task.

**Table 2:** General characteristics of the study participants according to *LPL* rs17482753 genotypes.

Variable	Men (*n* = 2558)			Women (*n* = 2433)		
	*LPL* rs17482753 Genotype					
	GG	TG, TT	*p*-Value ^(1)^	GG	TG, TT	*p*-Value ^(1)^
Participants, *n*	1951	607		1834	599	
Age (years)	51.3 ± 8.8	51.6 ± 8.8	0.55	50.1 ± 8.5	51.2 ± 8.7	0.01
Area			0.82			0.05
Ansung	814 (41.7%)	250 (41.2%)		774 (42.2%)	280 (46.7%)	
Ansan	1137 (58.3%)	357 (58.8%)		1060 (57.8%)	319 (53.3%)	
Body mass index (kg/m²)	23.4 ± 2.6	23.6 ± 2.8	0.08	23.9 ± 2.9	24.0 ± 2.9	0.6
Education level			0.29			0.42
≤Elementary school	384 (19.7%)	109 (18.0%)		624 (34.0%)	224 (37.4%)	
Middle school	406 (20.7%)	132 (21.8%)		477 (26.0%)	154 (25.7%)	
High school	715 (36.7%)	243 (40.0%)		589 (32.1%)	181 (30.2%)	
≥College	446 (22.9%)	123 (20.2%)		144 (7.9%)	40 (6.7%)	
Alcohol consumption (g/day)	18.2 ± 27.0	16.9 ±26.1	0.31	1.4 ± 5.1	1.1 ± 3.4	0.37
Energy (kcal/day)	1991.4 ± 566.0	1999.4 ± 548.4	0.76	1865.1 ± 594.4	1878.9 ± 643.2	0.64
Carbohydrate (g/day)	344.5 ± 93.9	345.7 ± 88.9	0.78	331.1 ± 103.9	334.8 ± 113.0	0.49
Protein (g/day)	68.4 ± 24.8	68.8 ± 25.8	0.70	63.5 ± 25.2	63.6 ± 26.4	0.93
Fat (g/day)	35.3 ± 18.3	35.4 ± 18.0	0.91	30.0 ± 17.2	30.0 ± 16.9	0.99
Smoking status			0.02			0.24
Never	383 (19.6%)	115 (19.0%)		1753 (95.6%)	578 (96.5%)	
Past	565 (29.0%)	211 (34.8%)		18 (1.0%)	8 (1.3%)	
Current	1003 (51.4%)	281 (46.2%)		63 (3.4%)	13 (2.2%)	
MET (hours/day) ^(2)^	25.0 ± 15.2	23.5 ± 14.6	0.03	22.1 ± 13.5	22.0 ± 14.5	0.93
Family history of diabetes			0.02			0.56
Yes	190 (9.7%)	79 (13.0%)		231 (12.6%)	70 (11.7%)	
No	1761 (90.3%)	528 (87.0%)		1603 (87.4%)	529 (88.3%)	
Marital status			0.10			0.01
Single	68 (3.5%)	30 (4.9%)		205 (11.2%)	90 (15.0%)	
Married	1883 (96.5%)	577 (95.1%)		1629 (88.8%)	509 (85.0%)	

Data are presented as means ± standard deviation (SD) or number (%). ^(1)^ *p*-values were calculated using the chi-squared test for categorical variables and the *t*-test for continuous variables. ^(2)^ MET, metabolic equivalent task.

**Table 3:** Association between total seaweed intake and the incidence of metabolic syndrome.

Total Seaweed								
Men (*n* = 2558)	Quartile 1		Quartile 2		Quartile 3		Quartile 4	
Multivariable HR (95% CI)	HR (95% CI)	*p*-value	HR (95% CI)	*p*-value	HR (95% CI)	*p*-value	HR (95% CI)	*p*-value
MetS	1.00 (Ref)		0.96 (0.81–1.13)	0.59	0.97 (0.81–1.16)	0.76	0.82 (0.69–0.99)	0.03
**Women (*n* = 2433)**	**Quartile 1**		**Quartile 2**		**Quartile 3**		**Quartile 4**	
Multivariable HR (95% CI)	HR (95% CI)	*p*-value	HR (95% CI)	HR (95% CI)	HR (95% CI)	*p*-value	HR (95% CI)	*p*-value
MetS	1.00 (Ref)		0.83 (0.70–0.98)	0.03	0.94 (0.79–1.12)	0.46	1.05 (0.89–1.24)	0.59

HR, hazard ratio; CI, confidence interval. Adjusted for age, area, alcohol consumption, smoking, body mass index, education level, family history of diabetes, marital status, and metabolic equivalents of task (MET).

**Table 4:** Association between *LPL* rs17482753 genotypes and the incidence of metabolic syndrome.

*LPL* rs17482753 Genotype				
Men (*n* = 2558)	GG		TG, TT	
Multivariable HR (95% CI)	HR (95% CI)	*p*-value	HR (95% CI)	*p*-value
Metabolic syndrome	1.00 (Ref)		0.83 (0.71–0.95)	0.01
Abdominal obesity	1.00 (Ref)		0.97 (0.82–1.15)	0.75
High blood pressure	1.00 (Ref)		0.79 (0.67–0.93)	0.004
High fasting glucose	1.00 (Ref)		1.09 (0.94–1.27)	0.24
High triglyceride levels	1.00 (Ref)		0.83 (0.70–0.99)	0.0471
Low HDL cholesterol levels	1.00 (Ref)		0.81 (0.69–0.95)	0.01
**Women (*n* = 2433)**	**GG**		**TG, TT**	
Multivariable HR (95% CI)	HR (95% CI)	*p*-value	HR (95% CI)	*p*-value
Metabolic syndrome	1.00 (Ref)		0.89 (0.78–1.03)	0.12
Abdominal obesity	1.00 (Ref)		0.97 (0.82–1.16)	0.76
High blood pressure	1.00 (Ref)		1.01 (0.85–1.21)	0.89
High fasting glucose	1.00 (Ref)		0.99 (0.82–1.18)	0.88
High triglyceride levels	1.00 (Ref)		0.85 (0.71–1.02)	0.08
Low HDL cholesterol levels	1.00 (Ref)		1.01 (0.85–1.21)	0.89

HR, hazard ratio; CI, confidence interval. Adjusted for age, area, alcohol consumption, smoking, body mass index, education level, family history of diabetes, marital status, and metabolic equivalents of task (MET).

**Table 5:** Association between *LPL* rs17482753 genotypes and the incidence of metabolic syndrome, stratified by total seaweed intake.

Total Seaweed									
Men (*n* = 2558)	Quartile 1		Quartile 2		Quartile 3		Quartile 4		*p*-Interaction
Multivariable HR (95% CI)	HR (95% CI)	*p*-value	HR (95% CI)	*p*-value	HR (95% CI)	*p*-value	HR (95% CI)	*p*-value	
GG	1.00 (Ref)		0.94 (0.77–1.13)	0.49	0.98 (0.80–1.20)	0.87	0.87 (0.71–1.06)	0.16	0.16
TG, TT	0.87 (0.66–1.16)	0.35	0.87 (0.66–1.15)	0.32	0.81 (0.60–1.09)	0.16	0.57 (0.41–0.79)	0.001	
**Women (*n* = 2433)**	**Quartile 1**		**Quartile 2**		**Quartile 3**		**Quartile 4**		***p*-Interaction**
Multivariable HR (95% CI)	HR (95% CI)	*p*-value	HR (95% CI)	*p*-value	HR (95% CI)	*p*-value	HR (95% CI)	*p*-value	
GG	1.00 (Ref)		0.92 (0.76–1.12)	0.39	1.01 (0.83–1.24)	0.92	1.09 (0.90–1.32)	0.40	0.52
TG, TT	1.08 (0.85–1.39)	0.52	0.66 (0.49–0.89)	0.01	0.82 (0.60–1.11)	0.20	1.03 (0.78–1.36)	0.85	

HR, hazard ratio; CI, confidence interval. Adjusted for age, area, alcohol consumption, smoking, body mass index, education level, family history of diabetes, marital status, and metabolic equivalents of task (MET).

## Data Availability

The dataset used in this study (Ansan–Ansung Cohort Study of the KoGES) was obtained after review and evaluation of the research plan of the National Biobank of Korea, Korea Disease Control and Prevention Agency (http://biobank.nih.go.kr (accessed on 20 August 2022).

## Supplementary Materials

The following are available online at https://www.mdpi.com/article/10.3390/nu15092066/s1, Supplementary Table S1: Association between laver intake and metabolic syndrome incidence Supplementary Table S2: Association between kelp and sea mustard intake and incidence of metabolic syndrome. Supplementary Table S3: Associations between *LPL* rs17482753 genotypes and the incidence of metabolic syndrome stratified by laver intake. Supplementary Table S4: Association between *LPL* rs17482753 genotypes and the incidence of metabolic syndrome stratified by kelp and sea mustard intake.

## Abbreviations

MetS, metabolic syndrome; *LPL*: lipoprotein lipase; CVD, cardiovascular disease; TC, total cholesterol; TG, triglyceride; LDL-C, low-density lipoprotein cholesterol; HDL-C, high-density lipoprotein cholesterol; KoGES, Korean Genome and Epidemiology Study; SQ-FFQ, semi-quantitative food frequency questionnaire; KARE, Korean Association Resource; CI, confidence interval; HR, hazard ratio; MET, metabolic equivalent; BMI: body mass index; NCEP-ATP III, National Cholesterol Educational Program Adult Treatment Panel III; OR: odds ratio.
