# Supplementary material for: Effect of the Interaction between Seaweed Intake and LPL Polymorphisms on Metabolic Syndrome in Middle-Aged Korean Adults
Source: Nutrients. 2023 Apr 25;15(9):2066. doi: 10.3390/nu15092066 (PMC10181071; doi:10.3390/nu15092066)
Supplement: Supplementary file 1 [file nutrients-15-02066-s001.zip › nutrients-2337617-supplementary.pdf]

Supplementary Table S1. Association between laver intake and metabolic syndrome (MetS) incidence.

Laver (grams/day)								
**Men (*n* = 2558)**	**Quartile 1**		**Quartile 2**		**Quartile 3**		**Quartile 4**	
Multivariable HR (95% CI)	HR (95% CI)	*P*-value	HR (95% CI)	*P*-value	HR (95% CI)	*P*-value	HR (95% CI)	*P*-value
MetS	1.00 (Ref)		1.07 (0.90-1.27)	0.46	0.90 (0.77-1.07)	0.23	0.83 (0.69-0.99)	0.04
**Women (*n* = 2433)**	**Quartile 1**		**Quartile 2**		**Quartile 3**		**Quartile 4**	
Multivariable HR (95% CI)	HR (95% CI)	*P*-value	HR (95% CI)	HR (95% CI)	HR (95% CI)	*P*-value	HR (95% CI)	*P*-value
MetS	1.00 (Ref)		0.99 (0.83-1.17)	0.87	1.02 (0.85-1.21)	0.86	1.08 (0.91-1.27)	0.40

1) HR, hazard ratio; CI, confidence interval
2) Adjusted for age, area, alcohol consumption, smoking, body mass index, education level, family history of diabetes, marital status, and metabolic equivalents of task (MET).

1)Adjusted for age, area, alcohol consumption, smoking, body mass index, education level, family history of diabetes, marital status, and metabolic equivalents of task (MET).

Supplementary Table S2. Association between kelp and sea mustard intake and incidence of metabolic syndrome (MetS).

Kelp/Sea mustard (grams/day)								
**Men (*n* = 2558)**	**Quartile 1**		**Quartile 2**		**Quartile 3**		**Quartile 4**	
Multivariable HR (95% CI)	HR (95% CI)	*P*-value	HR (95% CI)	*P*-value	HR (95% CI)	*P*-value	HR (95% CI)	*P*-value
MetS	1.00 (Ref)		1.20 (1.00-1.45)	0.05	1.04 (0.85-1.26)	0.71	1.04 (0.86-1.25)	0.70
**Women (*n* = 2433)**	**Quartile 1**		**Quartile 2**		**Quartile 3**		**Quartile 4**	
Multivariable HR (95% CI)	HR (95% CI)	*P*-value	HR (95% CI)	*P*-value	HR (95% CI)	*P*-value	HR (95% CI)	*P*-value
MetS	1.00 (Ref)		0.93 (0.78-1.10)	0.39	1.04 (0.89-1.23)	0.61	1.13 (0.94-1.35)	0.20

3) HR, hazard ratio; CI, confidence interval
1) Adjusted for age, area, alcohol consumption, smoking, body mass index, education level, family history of diabetes, marital status, and metabolic equivalents of task (MET).

Supplementary Table S3. Associations between *LPL* rs17482753 genotypes and the incidence of metabolic syndrome stratified by laver intake.

Laver (grams/day)									
**Men (*n* = 2558)**	**Quartile 1**		**Quartile 2**		**Quartile 3**		**Quartile 4**		***P*-interaction**
Multivariable HR (95% CI)	HR (95% CI)	*P*-value[1)]	HR (95% CI)	*P*-value[1)]	HR (95% CI)	*P*-value[1)]	HR (95% CI)	*P*-value[1)]	
GG	1.00 (Ref)		1.06 (0.87-1.29)	0.59	0.86 (0.72-1.04)	0.12	0.84 (0.68-1.02)	0.08	0.97
TG, TT	0.78 (0.60-1.03)	0.08	0.87 (0.65-1.16)	0.35	0.80 (0.61-1.05)	0.11	0.60 (0.43-0.84)	0.0029	
**Women (*n* = 2433)**	**Quartile 1**		**Quartile 2**		**Quartile 3**		**Quartile 4**		***P*-interaction**
Multivariable HR (95% CI)	HR (95% CI)	*P*-value[1)]	HR (95% CI)	*P*-value[1)]	HR (95% CI)	*P*-value[1)]	HR (95% CI)	*P*-value[1)]	
GG	1.00 (Ref)		1.05 (0.86-1.27)	0.66	1.02 (0.84-1.25)	0.84	1.03 (0.85-1.25)	0.74	0.26

TG, TT	0.90 (0.69-1.18)	0.45	0.78 (0.59-1.03)	0.08	0.92 (0.69-1.23)	0.58	1.11 (0.84-1.46)	0.46	

[1] HR, hazard ratio; CI, confidence interval
[2] Adjusted for age, area, alcohol consumption, smoking, body mass index, education level, family history of diabetes, marital status, and metabolic equivalents of task (MET).

Supplementary Table S4. Association between *LPL* rs17482753 genotypes and the incidence of metabolic syndrome stratified by kelp and sea mustard intake.

	**Kelp / Sea mustard (grams/day)**								
**Men (*n* = 2558)**	**Quartile 1**		**Quartile 2**		**Quartile 3**		**Quartile 4**		***P*-interaction**
Multivariable HR (95% CI)	HR (95% CI)	*P*-value[1]	HR (95% CI)	*P*-value[1]	HR (95% CI)	*P*-value[1]	HR (95% CI)	*P*-value[1]	
GG	1.00 (Ref)		1.23 (0.99-1.52)	0.06	0.99 (0.79-1.23)	0.90	1.03 (0.84-1.27)	0.77	0.57
TG, TT	0.87 (0.61-1.25)	0.45	1.00 (0.75-1.33)	0.98	0.92 (0.67-1.27)	0.63	0.77 (0.59-1.02)	0.07	
**Women (*n* = 2433)**	**Quartile 1**		**Quartile 2**		**Quartile 3**		**Quartile 4**		***P*-interaction**
Multivariable HR (95% CI)	HR (95% CI)	*P*-value[1]	HR (95% CI)	*P*-value[1]	HR (95% CI)	*P*-value[1]	HR (95% CI)	*P*-value[1]	
GG	1.00 (Ref)		1.00 (0.82-1.22)	0.99	1.07 (0.89-1.29)	0.45	1.12 (0.91-1.39)	0.29	0.17
TG, TT	1.18 (0.92-1.51)	0.19	0.81 (0.60-1.08)	0.14	0.75 (0.55-1.01)	0.05	1.13 (0.83-1.53)	0.45	

[1] HR, hazard ratio; CI, confidence interval
[2] Adjusted for age, area, alcohol consumption, smoking, body mass index, education level, family history of diabetes, marital status, and metabolic equivalents of task (MET).
